# *Ginkgo biloba* extract prescriptions are associated with less frequent repeat visits to ENT doctors due to tinnitus: a retrospective cohort study

**DOI:** 10.3389/fneur.2024.1402978

**Published:** 2024-07-31

**Authors:** Berthold Langguth, Thorsten Reineke, Martin Burkart, Karel Kostev

**Affiliations:** ^1^Department of Psychiatry and Psychotherapy, University of Regensburg, Regensburg, Germany; ^2^Research & Development, Dr. Willmar Schwabe GmbH & Co. KG, Karlsruhe, Germany; ^3^Global Medical Affairs, Dr. Schwabe Holding SE & Co. KG, Karlsruhe, Germany; ^4^IQVIA, Frankfurt am Main, Germany

**Keywords:** tinnitus, retrospective study, *Ginkgo biloba* extract, systemic corticosteroids, pentoxifylline, Germany, real-world data

## Abstract

**Objectives:**

We aimed to evaluate the drug utilization of *Ginkgo biloba* extract (Gbe), systemic corticosteroids (CSs), and pentoxifylline (PTXF) for the treatment of acute tinnitus by analyzing electronic patient health record data. In addition, we assessed whether the different drug treatments were associated with different frequencies of repeat visits to ear, nose, and throat (ENT) doctors.

**Methods:**

This retrospective cohort study used data from the IQVIA Disease Analyzer (DA) database. It included patients with an initial diagnosis of tinnitus between January 2005 and December 2021, treated by ENT specialists in Germany.

**Results:**

Of 111,629 patients meeting all selection criteria, 51,205 received prescriptions of Gbe, 34,817 of systemic CSs, and 25,607 of PTXF. Gbe prescription was associated with significantly lower odds of a repeat consultation due to tinnitus compared to systemic CSs [odds ratio (OR) 0.91; 95% confidence interval (CI): 0.88–0.95] as well as PTXF (OR 0.74; 95% CI: 0.72–0.77). This association was significant in both men and women and in some age groups.

**Conclusion:**

Gbe is the most frequently ENT specialist-prescribed drug for the treatment of acute tinnitus. It is associated with a reduced likelihood of patients consulting their ENT specialist for tinnitus again compared to systemic CSs and PTXF.

## 1 Introduction

Subjective tinnitus aurium describes the perception of sound in the absence of a corresponding external source. Sufferers hear unspecified acoustic sounds, such as ringing, buzzing, clicking, or pulsations, which may lead to tinnitus-related distress, i. e., by hindering sleep or concentration. The condition often has a recurrent or chronic course which can last for many years (1). Acute single-episode or intermittent tinnitus is defined as lasting up to 3 months, and chronic tinnitus is defined as lasting longer than 3 months (2). Impairments in chronic tinnitus are closely connected with psychosomatic and other concomitant symptoms and may reduce the overall quality of life (3). Furthermore, tinnitus-related economic burden is significant. In Germany, e.g., health-related costs sum up to 4798.91 EUR per year and per patient, according to conservative estimates (4).

Tinnitus is a common condition in Western countries. A meta-analysis of 83 publications revealed that among adults, the pooled prevalence was 14.4% for any tinnitus and 9.8% for chronic tinnitus (5). The European Tinnitus Survey conducted in 12 countries determined the prevalence of any tinnitus at 14.7% and of severe tinnitus at 1.2% (6). In Germany, 11.9% of people experienced tinnitus, for 5.5% of them, it was bothersome, and for 1%, it was severe. Tinnitus prevalence significantly increases with age and worsens hearing status (6).

For chronic idiopathic tinnitus, German and international treatment guidelines do not recommend any pharmacological treatments (7–9). Drug therapies for concomitant conditions such as depression or anxiety may lead to improvements, although they are not effective against tinnitus itself (9). Tinnitus is associated with hearing deficits in up to 90% of patients with chronic tinnitus, and hearing aids can improve tinnitus-related distress (10). General therapeutic counseling emphasizes coping strategies and stress reduction. Other non-pharmacological treatments include specific psychotherapy, neuromodulation, and sound therapy, albeit with often inconclusive evidence for efficacy (11). Digital tinnitus-related counseling is a new treatment approach currently under investigation (12). In Germany, no treatment guidelines for the treatment of acute tinnitus are available.

*Ginkgo biloba* special extracts such as EGb 761^®^ are registered in Germany and other countries for symptomatic or adjuvant therapy for patients with tinnitus. Systemic CSs are frequently prescribed for acute tinnitus (13), although there is no evidence for their efficacy (11). The vasoactive substance PTXF as an infusion or as short-term oral medication is also often prescribed. The latter have no marketing authorization for the treatment of tinnitus in Germany. For a long time, statutory health insurance reimbursed the costs of CS and PTXF therapy for sudden sensorineural hearing loss, but not for tinnitus.

The importance of real-world data is increasingly recognized as a source of medical and scientific insights. Both prospective and retrospective data collection can be analyzed. Real-world studies allow for the investigation of diverse, large, and heterogeneous patient populations. These studies are characterized by high ecological validity as they are not subject to the patient selection bias that can occur in randomized controlled trials, which typically apply stringent patient eligibility criteria. Randomized controlled trials, which are the gold standard to assess the efficacy of a drug, should therefore be supplemented by real-world data due to the better reflection of the situation in daily routine practice. This study analyzes the prevalence and effects of drug prescriptions of Gbe, CS, and PTXF in patients with newly diagnosed tinnitus. The primary outcome criterion as a surrogate for efficacy was the frequency of repeat consultations. The analysis was based on an electronic health record database with datasets of patients, representing a large and diverse patient population. Moreover, for some patients, data covering a period of as long as 16 years were available, allowing the longitudinal analysis of outcomes.

## 2 Methods

### 2.1 Data source

This analysis was performed with the IQVIA DA database, which contains patient information provided by office-based physicians in Germany. These are more than 3,500 physicians, general practitioners, and various specialists, including approximately 200 ear, nose, and throat (ENT) specialists, representing approximately 3% of all German doctor’s practices. The sample covers eight geographical regions and is representative of Germany. The database appears to be suitable for pharmacoepidemiological or pharmacoeconomic studies (14).

The DA contains datasets of more than 10 million patients. Each dataset consists of patient demographics, diagnoses, drug prescriptions (including the diagnosis for which a specific drug was prescribed), concomitant medication, comorbid conditions, sick leave, and hospital referrals. IQVIA ensures the accuracy, consistency, and completeness of the data through regular monitoring.

Data analysis was performed in compliance with German legislation for the use of anonymous de-identified electronic medical records for research purposes.

### 2.2 Study population

All study definitions were *a priori* defined and summarized in the study protocol prior to analysis. This study included patients aged 16 years or older with a diagnosis of tinnitus as coded with the International Statistical Classification of Diseases and Related Health Problems version 10 (ICD-10) H93.1. Patients with at least one prescription of Gbe (herbal medicinal products), CS, or PTXF within 2 weeks after tinnitus diagnosis were considered. Analysis was restricted to data provided by specialized physicians for ENT diseases and entered into the database between January 2005 and December 2021. Patients were excluded when they had received more than one of the three drugs within 2 weeks after the tinnitus diagnosis or when information on age or sex was missing. The 2-week criterion was considered the time between tinnitus diagnosis and first prescription and was ≤14 days in more than 99% of study patients.

The coding with ICD-10 does not differentiate between acute and chronic tinnitus. However, because of the 2-week criterion after tinnitus diagnosis, it was assumed that the majority of participants suffered from acute tinnitus.

The population was described using patient age, sex, insurance status (private or statutory), and idiopathic sudden sensorineural hearing loss (ICD-10 H91.2), otitis media (ICD-10 H65, H66, H67), or disorders of vestibular function (ICD-10: H81) as concomitant conditions. Since ENT specialists rarely diagnose non-otolaryngological diseases such as depression or diabetes, these concomitant conditions were not included as covariables in the analysis.

### 2.3 Outcomes and statistical analyses

In the present study, patients were followed from day 15 after their first tinnitus diagnosis until the first repeated consultation of the same ENT specialist due to tinnitus, or—in the absence of a repeat consultation—until day 365 after the first prescription. In a sensitivity analysis, only patients with a prescription on the day of tinnitus diagnosis were included and then followed from day 1 after their first tinnitus diagnosis.

A multivariable logistic regression analysis was performed to compare the effect of Gbe prescriptions with prescriptions of CS and PTXF, respectively, on repeated visits to the same ENT specialist due to tinnitus. Regression analyses were adjusted to age, sex, health insurance coverage, and co-diagnoses (idiopathic sudden sensorineural hearing loss, otitis media, and disorders of vestibular function).

The results of the logistic regression analysis were displayed in the form of ORs with 95% CIs. ORs were shown for Gbe therapy, adjusted for the defined confounders.

Regression models were run separately for male and female patients and for six age groups (≤30, 31–40, 41–50, 51–60, 61–70, and > 70 years). A *p*-value of <0.05 was considered statistically significant.

## 3 Results

**Figure 1:**
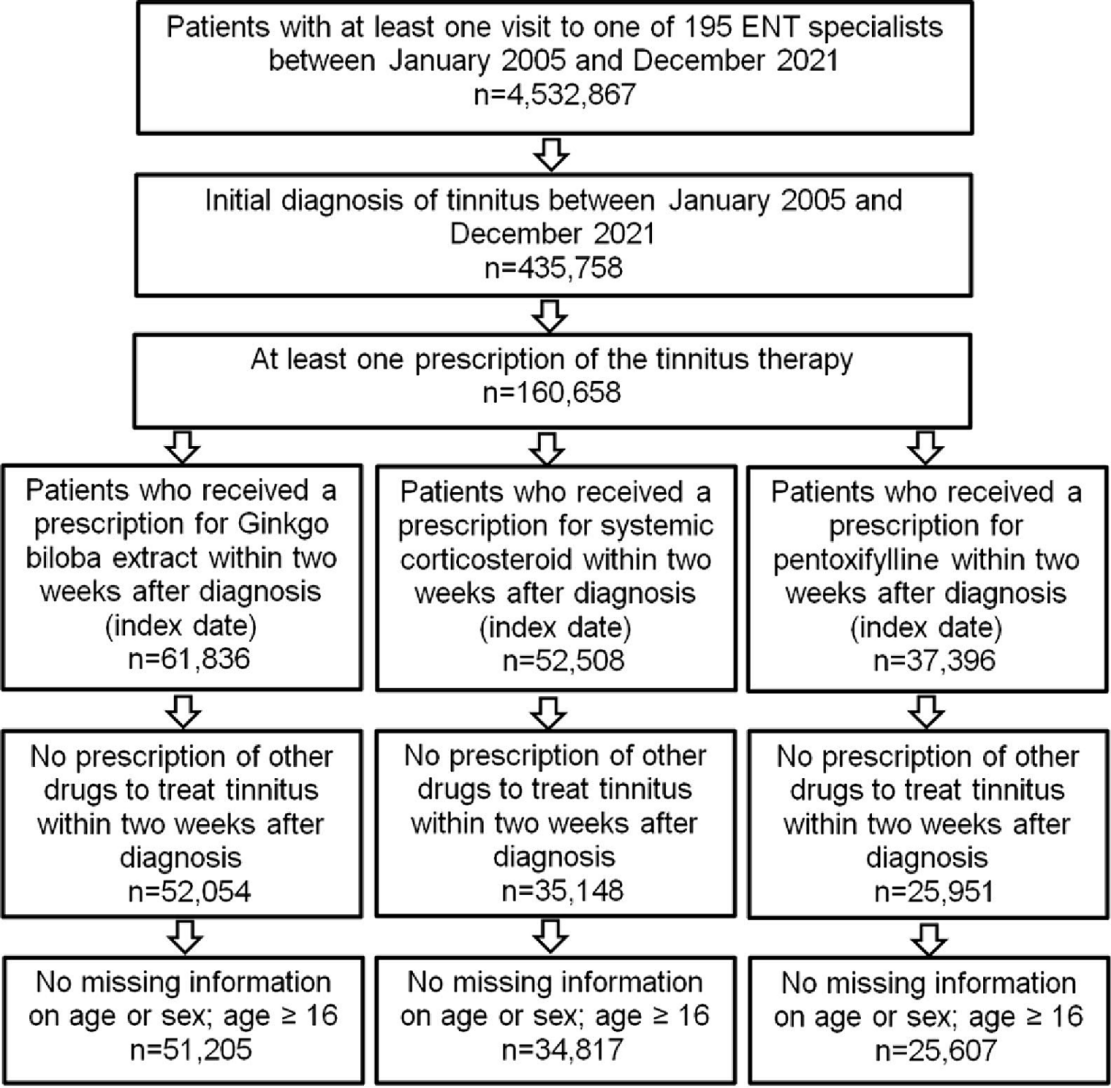
Selection of study patients.

More than 4.5 million patient visits to ENT specialists from 2005 to 2021 were documented in the IQVIA DA database. Of them, 435,758 patients were diagnosed with tinnitus, and 160,658 received at least one prescription for pharmacological treatment of their tinnitus. Gbe was prescribed to 61,836 (14.2%), CS to 52,508 (12.0%), and PTXF to 37,396 (8.6%) of them. Other drugs, including betahistine, acetylsalicylic acid, and homeopathic products, were prescribed to only small proportions of patients. In total, 51,205 Gbe, 34,817 CS, and 25,607 PTXF patients met all selection criteria, representing the three most frequently prescribed tinnitus therapies in ENT practices (Figure 1). A total of 93.0% of patients received the first prescription on the day of diagnosis, 2.2% on days 1–3, 1.6% on days 4–6, 1.6% on days 7–9, and 1.6% on days 10–14 after the diagnosis date.

### 3.1 Baseline characteristics

Table 1 shows the baseline characteristics of the included patients. Patients receiving Gbe prescriptions were slightly older (52.9 years) than those receiving CS (47.4 years) or PTXF (49.4 years). The proportion of female patients was significantly higher in the Gbe prescription group (55.0%) than in CS (47.0%) or PTXF (47.7%). The three cohorts differed slightly regarding the percentage of patients with private insurance coverage (Gbe: 14.6%, CS: 12.5%, PTXF: 11.0%). The biggest difference between the three cohorts was concomitant idiopathic sudden sensorineural hearing loss, which was diagnosed in 19.8% for Gbe compared to 37.4% for CS and 25.3% for PTXF.

**Table 1:** Basic characteristics of study patients.

Variable	Patients with *Ginkgo biloba* extract prescription (N, %)	Patients with systemic corticosteroid prescription (N, %)	Patients with pentoxifylline prescription (%)	*p*-value (comparison between three cohorts)
N	51,205	34,817	25,607	
Age (mean, SD)	52.9 (16.5)	47.7 (15.2)	49.4 (15.5)	<0.001
≤30 years	5,707 (11.2)	5,442 (15.6)	3,433 (13.4)	<0.001
31–40 years	6,361 (12.4)	5,991 (17.2)	3,941 (15.4)	
41–50 years	9,631 (18.8)	8,022 (23.0)	5,995 (23.4)	
51–60 years	12,310 (24.0)	8,350 (24.0)	5,865 (22.9)	
61–70 years	9,080 (17.7)	4,398 (12.6)	3,883 (15.2)	
>70 years	8,116 (15.9)	2,614 (7.5)	2,490 (9.7)	
Sex: female	28,142 (55.0)	16,377 (47.0)	12,215 (47.7)	<0.001
Sex: male	23,063 (45.0)	18,440 (53.0)	13,392 (52.3)	
Statutory health insurance coverage	43,751 (85.4)	30,468 (87.5)	22,777 (89.0)	<0.001
Private health insurance coverage	7,454 (14.6)	4,349 (12.5)	2,830 (11.0)	<0.001
Idiopathic sudden sensorineural hearing loss	9,119 (17.8)	12,716 (36.5)	6,074 (23.7)	<0.001
Otitis media	3,523 (6.9)	3,197 (9.2)	2,015 (7.9)	
Disorders of vestibular function	1,669 (3.3)	1,642 (4.7)	745 (2.9)	

**Figure 2:**
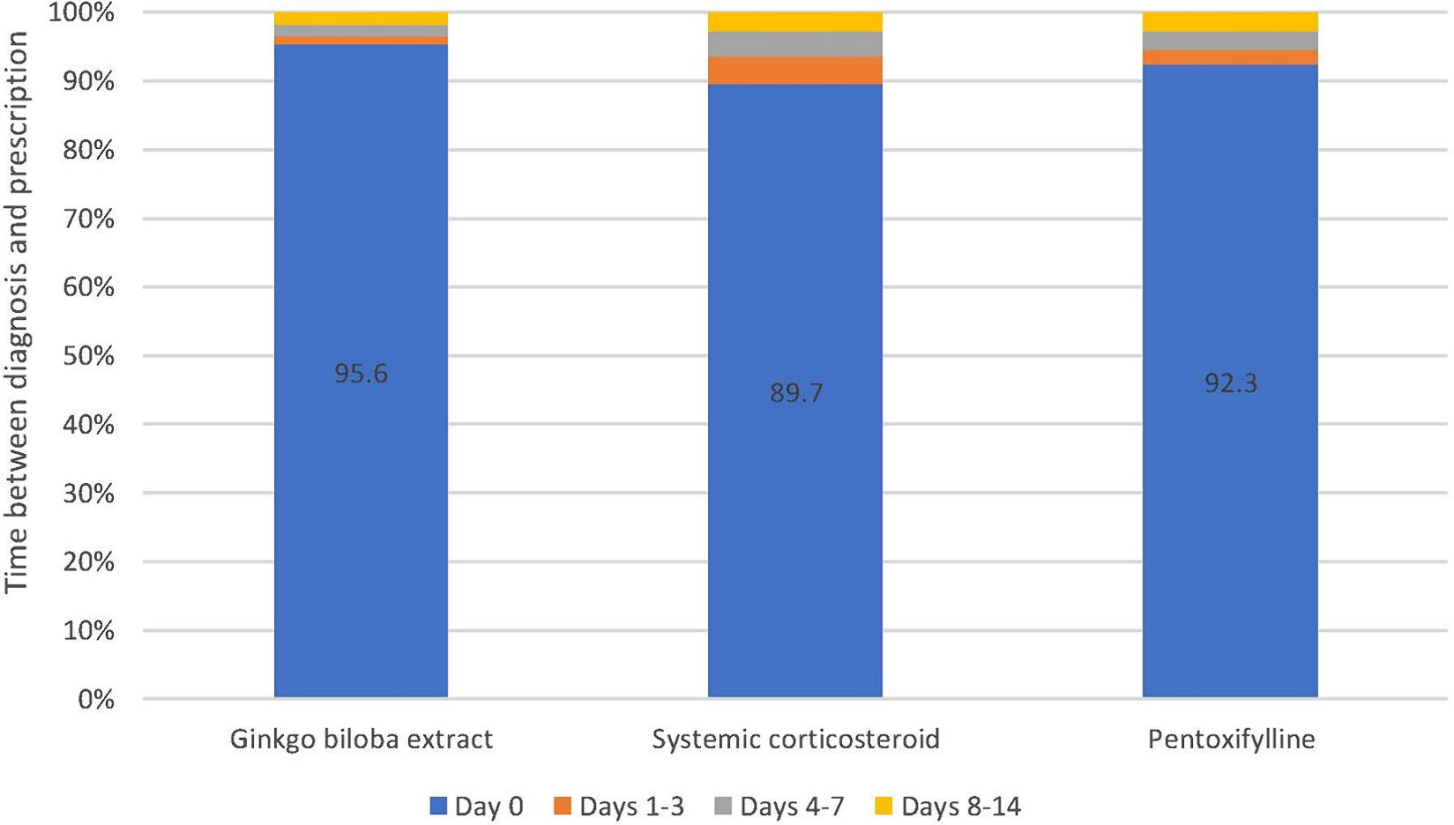
Time interval between tinnitus diagnosis and first prescription of the study treatments.

Most patients received their first prescription on the day when the diagnosis was set (Gbe: 95.6%, CS: 89.7%, PTXF: 92.3%). The proportion of patients who received the first prescription later was rather small (Figure 2).

### 3.2 Association between Gbe prescriptions and repeat visits due to tinnitus

Table 2 shows the proportion of patients in the Gbe and CS cohorts, who consulted the same ENT specialist again due to tinnitus during the period 15–365 days after treatment initiation, as well as the results of the multivariable regression analysis stratified by age group and sex.

**Table 2:** Association between *Ginkgo biloba* extract (Gbe) prescription compared to systemic corticosteroid (CS) prescription and repeat consultation due to tinnitus within 15–365 days after therapy initiation.

Patient group	Proportion of patients with reconsultation after Gbe prescription (%)	Proportion of patients with reconsultation after CS prescription (%)	Odds ratio (95% CI)	*P*-value
Total*	22.5	23.7	0.91 (0.88–0.95)	<0.001
≤30 years**	15.5	16.4	0.97 (0.87–1.07)	0.521
31–40 years**	18.3	21.3	0.86 (0.78–0.94)	<0.001
41–50 years**	23.3	25.6	0.91 (0.84–0.97)	0.006
51–60 years**	25.1	26.8	0.95 (0.89–1.01)	0.126
61–70 years**	25.4	27.1	0.94 (0.87–1.03)	0.177
>70 years**	22.3	23.0	0.97 (0.87–1.07)	0.488
Women***	22.2	23.9	0.89 (0.85–0.94)	<0.001
Men***	22.8	23.5	0.93 (0.89–0.98)	0.005

*Multivariable logistic regression adjusted for age, sex, health insurance coverage, and co-diagnoses (idiopathic sudden sensorineural hearing loss, otitis media, and disorders of vestibular function). **Multivariable logistic regression adjusted for sex, health insurance coverage, and co-diagnoses (idiopathic sudden sensorineural hearing loss, otitis media, and disorders of vestibular function). ***Multivariable logistic regression adjusted for age, health insurance coverage, and co-diagnoses (idiopathic sudden sensorineural hearing loss, otitis media, and disorders of vestibular function).

A repeated consultation within 15–365 days after treatment initiation was documented in 22.5% of patients after the Gbe prescription and in 23.7% of patients after the CS prescription. Gbe prescription was associated with significantly lower odds of a repeat consultation (OR: 0.91; 95% CI: 0.88–0.95) after adjustment for age, sex, health insurance coverage, and co-diagnoses (idiopathic sudden sensorineural hearing loss, otitis media, and disorders of vestibular function). This association was observed in men and women, and in all age groups, although it was not significant in all of them (Table 2).

A repeated consultation within 15–365 days after treatment initiation was documented in 27.5% of patients after PTXF prescription. Gbe prescription was strongly associated with significantly lower odds of a repeat consultation (OR 0.74; 95% CI: 0.72–0.77). This association was significant in both men and women and in all age groups (Table 3).

**Table 3:** Association between *Ginkgo biloba* extract (Gbe) prescription compared to pentoxifylline (PTXF) prescription and repeat consultation due to tinnitus within 15–365 days after therapy initiation.

Patient group	Proportion of reconsultation after Gbe prescription (%)	Proportion of reconsultation after PTXF prescription (%)	Odds ratio (95% CI)	*P*-value
Total*	22.5	27.5	0.74 (0.72–0.77)	<0.001
≤30 years**	15.5	17.9	0.85 (0.76–0.95)	0.005
31–40 years**	18.3	24.7	0.69 (0.63–0.76)	<0.001
41–50 years**	23.3	29.0	0.75 (0.69–0.80)	<0.001
51–60 years**	25.1	31.9	0.72 (0.67–0.77)	<0.001
61–70 years**	25.4	29.8	0.81 (0.74–0.88)	<0.001
>70 years**	22.3	27.9	0.73 (0.66–0.81)	<0.001
Women***	22.2	26.8	0.76 (0.72–0.80)	<0.001
Men***	22.8	28.2	0.73 (0.69–0.76)	<0.001

*Multivariable logistic regression adjusted for age, sex, insurance coverage, and co-diagnoses (idiopathic sudden sensorineural hearing loss, otitis media, and disorders of vestibular function). **Multivariable logistic regression adjusted for sex, insurance coverage, and co-diagnoses (idiopathic sudden sensorineural hearing loss, otitis media, and disorders of vestibular function). ***Multivariable logistic regression adjusted for age, insurance coverage, and co-diagnoses (idiopathic sudden sensorineural hearing loss, otitis media, and disorders of vestibular function).

### 3.3 Sensitivity analysis

The results of the sensitivity analysis showed Gbe prescription to be strongly associated with significantly lower odds of repeat consultation within 1–365 days after treatment initiation compared to PTXF prescription (OR 0.63; 95% CI: 0.61–0.65) and CS prescription (OR 0.73; 95% CI: 0.70–0.75), respectively.

## 4 Discussion

This large-scale retrospective real-world study used electronic medical records as a data source. It was performed on the datasets of 111,629 tinnitus-diagnosed patients and delivered insights regarding the drug utilization of Gbe, CS, and PTXF. Gbe was the most frequently prescribed monotherapy at the first consultation of an ENT specialist for tinnitus. Our observations are in line with a previous survey where 70% of ENT specialists reported offering pharmaceutical treatments for acute tinnitus, while drugs were rarely offered for chronic tinnitus (15). Therefore, the large proportion of patients receiving drug prescriptions for acute tinnitus does not contradict guidelines for chronic tinnitus that do not recommend the prescription of drugs. One possible explanation for the high number of prescribed drugs is the expectation of tinnitus patients to receive pharmacological treatment for their tinnitus (16). Another explanation might be the professional experience of ENT specialists on the usefulness of these drugs in acute tinnitus.

In addition, the analysis shows that Gbe prescription was associated with a reduced likelihood of a patient consulting the same ENT specialist due to tinnitus again compared to CS and PTXF, even after controlling for confounders such as idiopathic sudden sensorineural hearing loss or insurance coverage. Due to the study’s observational nature, it does not allow conclusions if this was causally related.

Important concomitant conditions were unevenly distributed between groups: idiopathic sudden sensorineural hearing loss was twice as frequent in patients receiving CSs compared to patients receiving Gbe, and there were also differences in the prevalences of otitis media and disorders of vestibular function. The presence of such conditions can necessitate repeated ENT visits *per se* and can impact the course of tinnitus. Therefore, the comparison of the absolute repeat consultation rates is of limited informative value, and we adjusted logistic regression analyses for these co-diagnoses. Interpretation of results is based on adjusted ORs. An OR of 0.74 versus PTXF is clinically meaningful, while the OR of 0.91 versus CS is of borderline relevance. Both ORs indicate that Gbe treatment necessitates the lowest need for re-consultations. ENT visits contribute approximately 13% to the total tinnitus treatment costs in Germany. Our results indicate that tinnitus treatment with Gbe might be associated with a lower socioeconomic burden (4).

Previously published studies investigating the therapy of tinnitus with Gbe have shown mixed results A meta-analysis of three trials in patients with tinnitus as the primary complaint suggests the efficacy of the special extract EGb 761^®^ in the treatment of tinnitus (17). In a randomized controlled trial, EGb 761^®^ and PTXF were similarly effective in reducing tinnitus loudness and annoyance (18). In a small randomized double-blind trial, both EGb 761® and hearing aids resulted in a significant improvement in tinnitus loudness and severity (19). A small open-label study reported improvements in tinnitus with escalating doses of clonazepam but not with Ginkgo extract (20). Another small randomized trial did not observe the benefit of adding a methanolic St. John’s wort extract to EGb 761^®^, while tinnitus handicap improved with the *Ginkgo* extract (21). Supportive evidence was delivered by five trials in patients with age-associated cognitive impairment or dementia in whom tinnitus was present as a concomitant symptom (22). Direct effects on tinnitus accounted for 60% of the total effect (23). In a randomized trial, adding antioxidants to an unspecified Ginkgo preparation provided a marked improvement in THI, VAS, and SF-36 scores (24). Other trials using a different Gbe than EGb 761^®^ were unable to demonstrate a significant improvement in tinnitus (25, 26). A Cochrane review (27) concluded that there is uncertainty about the benefits and harms of *Ginkgo biloba* for the treatment of tinnitus when compared to placebo. The mixed results might be related to variable compositions of the extracts investigated in the different studies. Plant extracts, in general, are multi-constituent drugs. Constituents of Ginkgo leaf extracts, such as terpene lactones, flavone glycosides, and proanthocyanidines, address multiple molecular and cellular pathways (28). Notably, extracts derived from the same plant that were manufactured by different production processes can have very different phytochemical compositions. The results from pharmacological, toxicological, and clinical studies are therefore specific to the investigated preparation and cannot necessarily be extrapolated from one to another (29).

Real-world studies have their merits as they include diverse and large patient populations with heterogeneous demographics, comorbidities, or comedications and better reflect the complexity of everyday medical practice. However, real-world data also have substantial shortcomings, e.g., the lack of a control group and a limitation in the type and quantity of outcome parameters. This study used the frequency of repeat visits as an outcome parameter. Many factors can influence the frequency of repeat consultations and one cannot conclude from a lower frequency of repeat visits to the efficacy of an intervention. Effective symptom reduction may reduce the frequency of repeat visits, but also dissatisfaction with the treatment, side effects, or even death. Moreover, further factors such as prescription status of treatments, package sizes, or insurance reimbursement may play a role. The actual German Gbe prescribing information stipulates that the adjuvant treatment of tinnitus should be carried out for at least 12 weeks. If no therapeutic success is observed after 6 months, no further improvement is to be expected after that. The prescribing information for PTXF does not stipulate a treatment duration or re-consultation, established treatment protocols recommended 10 days (30). CS is not approved for the treatment of tinnitus in Germany, variable treatment protocols have been used. In consequence, differences in clinical protocols might have contributed to the observed differences. Thus, real-world data cannot replace but only complement data from randomized controlled trials. On the other hand, real-world data provides insights into how treatments work in a large and therefore more representative sample of the population. Real-world studies can further assess the heterogeneity of treatment effects across different subpopulations, helping to identify which patients benefit most from a particular intervention (31, 32). In the present analysis, regression models were conducted separately for women and men as well as for age groups, and analyses were adjusted for all available comorbidities.

Retrospective analyses like ours are generally limited by the validity and completeness of the available data, which should be mentioned at this point. The analysis was limited to datasets of ENT specialists since they are mainly responsible for the diagnosis and treatment of tinnitus. However, they rarely document non-otolaryngological concomitant diseases, thus this information is missing. Concomitant conditions such as depression, anxiety disorder, stress-related conditions, or diabetes mellitus may be confounders for the outcome (3). Moreover, we have no information about potential tinnitus-related revisits by general practitioners or other ENT specialists. It is also possible that patients suffered from tinnitus symptoms some days or weeks prior to the first visit to the ENT specialist, as a significant number of patients in Germany often have to wait until the next free appointment. The duration of symptomatic tinnitus may impact the severity and the choice of therapy. In addition, patients can only be observed in a single practice; if they additionally receive a diagnosis or prescription from another practice, such prescriptions are not included in the database documentation. Furthermore, the diagnoses in the database are coded by ICD-10, which does not include an assessment of disease severity. However, it is unlikely that the severity of tinnitus was decisive for the choice of therapy (Gbe, CS, and PTXF) rather than, e.g., physician and/or patient preference. Due to the lack of a control group, only comparisons between different treatments were possible. Furthermore, there is no information about the dosage. With respect to CSs, a recent large randomized controlled trial suggests that CS at high doses might even worsen the outcome of patients with sudden hearing loss as compared to low doses of prednisolone (33). Moreover, no information is available on non-pharmacological therapies, which also might have an impact on the outcome. Furthermore, the database does not include data on the use of herbal medicines purchased without prescription. Due to the large datasets, however, it is assumed that these factors are equally distributed in the prescription cohorts. Finally, retrospective studies do not allow any conclusions to be drawn about the causal effects but only about statistical associations.

In conclusion, our study contributes to the knowledge of drug utilization for tinnitus management. It opens avenues for future research to elucidate the underlying mechanisms and confirm causality, ultimately enhancing our ability to provide effective interventions for patients suffering from tinnitus.

## Data availability statement

The data analyzed in this study is subject to the following licenses/restrictions: the datasets used and analyzed during the current study are available from the corresponding author on reasonable request. Requests to access these datasets should be directed to karel.kostev@iqvia.com.

## Ethics statement

Ethical approval was not required for the study involving humans in accordance with the local legislation and institutional requirements. Written informed consent to participate in this study was not required from the participants or the participants’ legal guardians/next of kin in accordance with the national legislation and the institutional requirements.

## Author contributions

BL: Conceptualization, Writing – review & editing. TR: Conceptualization, Writing – original draft. MB: Conceptualization, Writing – review & editing. KK: Conceptualization, Formal Analysis, Writing – original draft.
